# Human Liver Spheroids as a Model to Study Aetiology and Treatment of Hepatic Fibrosis

**DOI:** 10.3390/cells9040964

**Published:** 2020-04-14

**Authors:** Tracey Hurrell, Vlasia Kastrinou-Lampou, Achilleas Fardellas, Delilah F. G. Hendriks, Åsa Nordling, Inger Johansson, Audrey Baze, Céline Parmentier, Lysiane Richert, Magnus Ingelman-Sundberg

**Affiliations:** 1 Ingelman-Sundberg Group, Section of Pharmacogenetics, Department of Physiology and Pharmacology, Karolinska Institutet, 171 65 Stockholm, Sweden; THurrell@csir.co.za (T.H.); vlasia.kastrinou-lampou@ki.se (V.K.-L.); achilleas.fardellas@astrazeneca.com (A.F.); d.hendriks@hubrecht.eu (D.F.G.H.); asa.nordling@ki.se (Å.N.); Inger.Johansson@ki.se (I.J.); 2 KaLy-Cell, 67115 Plobsheim, France; a.baze@kaly-cell.com (A.B.); c.parmentier@kaly-cell.com (C.P.); l.richert@kaly-cell.com (L.R.)

**Keywords:** primary human hepatocytes, stellate cells, co-culture spheroids, COL1A1, CYP2E1, αSMA, ALK5 inhibitor

## Abstract

Non-alcoholic fatty liver disease affects approximately one billion adults worldwide. Non-alcoholic steatohepatitis (NASH) is a progressive disease and underlies the advancement to liver fibrosis, cirrhosis, and hepatocellular carcinoma, for which there are no FDA-approved drug therapies. We developed a hetero-cellular spheroid system comprised of primary human hepatocytes (PHH) co-cultured with crude fractions of primary human liver non-parenchymal cells (NPC) from several matched or non-matched donors, to identify phenotypes with utility in investigating NASH pathogenesis and drug screening. Co-culture spheroids displayed stable expression of hepatocyte markers (albumin, CYP3A4) with the integration of stellate (vimentin, PDGFRβ), endothelial (vWF, PECAM1), and CD68-positive cells. Several co-culture spheroids developed a fibrotic phenotype either spontaneously, primarily observed in PNPLA3 mutant donors, or after challenge with free fatty acids (FFA), as determined by COL1A1 and αSMA expression. This phenotype, as well as TGFβ1 expression, was attenuated with an ALK5 inhibitor. Furthermore, CYP2E1, which has a strong pro-oxidant effect, was induced by NPCs and FFA. This system was used to evaluate the effects of anti-NASH drug candidates, which inhibited fibrillary deposition following 7 days of exposure. In conclusion, we suggest that this system is suitable for the evaluation of NASH pathogenesis and screening of anti-NASH drug candidates.

## 1. Introduction

Environmentally and societally induced diseases are epidemic in their prevalence and require concentrated healthcare and pharmaceutical management. One such clinical burden is non-alcoholic fatty liver disease (NAFLD), a disease characterized by macro-vesicular steatosis of the liver in persons consuming little alcohol. Simple steatosis and non-alcoholic steatohepatitis (NASH) are the two major distinguishable NAFLD phenotypes [1,2]. NASH is the progressive form of NAFLD which underlies the development of fibrosis, cirrhosis, and hepatocellular carcinoma [3,4]. In 2013, the global prevalence of NAFLD approximated 1 billion individuals [3], with recent estimates reaching 25% in adults [5]. Clinically, NASH, and its advancement to fibrosis, remain an unmet medical liability as there are no FDA-approved drug therapies available. In addition to the pathophysiological complexities associated with progression, heritability is a contributor to the disease, where primarily genetic variants of Patatin-like phospholipase domain-containing protein 3 (PNPLA3) as well as Transmembrane 6 Superfamily Member 2 (TM6SF2) have been clinically evidenced [6].

Concerted efforts are being placed on elucidating the signaling pathways in NASH [7], with numerous Phase II and III clinical trials being undertaken [8]. However, potential anti-NASH drugs have shown limited efficacy throughout the clinical phases of development [9]. While there are murine models for each stage of NAFLD, translational limitations and discrepancies in their histopathology and/or physiological properties cannot be overlooked [10]. Accordingly, there is an urgent need to develop human-based in vitro models comprising the essential cellular lineages present in the liver which underlie the development of NASH. The cellular mechanisms involved in the initiation and progression of fibrosis are orchestrated by oxidative stress, which drives hepatocyte damage and non-parenchymal cell (NPC) activation, with transforming growth factor-β (TGF-β) being a central regulator in these processes [11]. Hepatic stellate cells (HSCs) undergo differentiation to a pro-fibrotic phenotype which is associated with liver fibrosis [12,13,14], making these NPCs integral to evaluating pharmaceutical interventions. Advances in tissue engineering support increased cellular longevity in vitro, which is achieved by a three-dimensional (3D) spatial arrangement of cells, and where applied to liver tissue, demonstrate improved hepatocyte viability and functionality [15,16,17]. Primary human hepatocytes (PHH) cultured as 3D spheroids maintain tissue-like architecture, cell–cell interactions, and hepatocyte phenotype [18,19], and have therefore successfully been used to model hepatotoxicity [20,21,22], cholestasis [23], steatosis and insulin resistance [24,25], as well as the impact of genetic variants on lipid biosynthesis [26]. Historically, in vitro liver cultures lacked hetero–cellular interactions, however, co-cultures improve hepatocyte functionality and allow the study of PHH–NPC interactions [27,28,29]. Furthermore, bio-printed or spheroid co-cultures which modulate extracellular matrix [30,31] and inflammatory responses [32,33] have provided promising proof-of-principle for modelling NAFLD but currently lack critical high-throughput compatibility. In this study, a high-throughput screening (HTS)-compatible human liver spheroid system was established, using commercially available donor PHH and NPCs, and applied to model the fibrotic aspects of NASH. This PHH spheroid system has previously been shown to mimic the human liver in vivo with respect to proteomics [18], transcriptomics, and metabolomics [19], with the feasibility of co-cultures previously demonstrated [32,33]. We characterized hetero-cellular spheroids from eight different donors with matched and non-matched, ratio controlled NPCs, and interrogated their use in modelling fibrotic phenotypes and applications for anti-NASH compound screening. We propose that this spheroid system is useful for the evaluation of mechanisms involved in NASH pathogenesis and for acute and chronic target-independent screening of compounds having anti-NASH properties.

## 2. Materials and Methods

### 2.1. Spheroid Cultures

Cryopreserved PHH and crude NPCs were obtained from Bioreclamation IVT (BioIVT, NY, USA), KaLy-Cell (KLC, Plobsheim, France), and Lonza (Basel, Switzerland). PHH were seeded, as previously described [18,33], into Corning 96-well ultra-low attachment plates with or without the addition of NPCs seeded at a 4:1 ratio of PHH:NPC (1500:375). Initial titration experiments, at ratios ranging from 2:1 to 6:1 using fixed numbers of PHH, were conducted. The ratio most amenable to reproducible incorporation of cells into the spheroid, which was also physiologically representative of the parenchymal to non-parenchymal cell ratio, was selected for further experiments (Supplementary Figure S1). Spheroids were seeded in Williams E medium supplemented with 2 mM L-glutamine, 100 units/mL penicillin, 100 μg/mL streptomycin, 100 nM dexamethasone, 10 μg/mL insulin, 5.5 μg/mL transferrin, 6.7 ng/mL sodium selenite, and 10% fetal bovine serum (Life Technologies, Thermo Fisher Scientific, MA, USA). Following aggregation, transition to serum-free medium was initiated. Six PHH and six NPC donors (Table 1) were used in donor-matched or non-matched configurations. NPCs from donors 1 to 4 were passaged, as previously described [33], over a period of 5–10 days prior to cryopreservation. Availability of cells from each donor ranged from 1 to 8 vials of PHH and/or NPCs, which limited the number of biological replicates and exposure to exogenous stimuli for some donors.

### 2.2. Functional Responses

Spheroids were exposed to 5 ng/mL exogenous transforming growth factor β1 (TGFβ1, 240-B-002; R&D systems, MN, USA) for 72 h on day 7, and the gene expression for TGFβ1, lysyl oxidase (LOX), and type I collagen (COL1A1) was determined. Modulation of exogenous TGFβ1 was assessed with a 1 h pre-exposure (50 μM) or continuous co-exposure (5 μM) to a TGFβ1 receptor inhibitor (TGFβ1Ri, ALK5 inhibitor: SB525334 (3211; Tocris Bioscience, Bristol, UK)). Furthermore, regulation of endogenous TGFβ1 expression was investigated with exposure to TGFβRi alone (0.5 and 5 μM) from seeding or day 7. Spheroids were also exposed to 1 µg/mL lipopolysaccharide (LPS, L6529-1MG; Sigma Aldrich, MO, USA) for 48 h on day 7 and the gene expression of interleukin-6 (IL-6) was determined.

### 2.3. Induction of a NASH-Like Phenotype

Spheroid monocultures and co-cultures were exposed to lipogenic substances, as previously described (25), with minor modifications. Unsaturated oleic acid and saturated palmitic acid (Sigma Aldrich, MO, USA), solubilized in ethanol, were conjugated to 10% bovine serum albumin at a 1:5 molar ratio for 2 h at 40 °C. The free fatty acids (FFA) were combined in a 1:1 ratio and spheroids were exposed to 480 µM from day 5 to 14 with medium exchanged every 2 to 3 days.

### 2.4. Drug Screening

Co-cultures of donor 1, which progressively and reproducibly developed a pro-fibrogenic phenotype, were used to screen anti-fibrotic compounds currently in Phases II and III of clinical development [34,35,36]. Spheroids were repeatedly exposed to cenicriviroc (HY-14882; MedChem Express, NJ, USA), elafibranor (HY-16737; MedChem Express, NJ, USA), or lanifibranor (HY-104049; MedChem Express, NJ, USA) from day 7 to 14. Drug concentrations were initially investigated at approximately 2 to 20 times the in vivo C_max_ (Supplementary Table S1) and titrated to non-toxic concentrations where required.

### 2.5. Cell Viability

ATP content was measured using a CellTiter Glo Luminescent Cell Viability Assay kit (Promega, WI, USA), as per the manufacturer’s instructions. Twenty-five microliters of reconstituted assay reagent was added per well containing 20 µL medium. Spheroids were mechanically disrupted by pipetting and the plate was incubated at 37 °C for 30 min. The luminescence signal was measured using a MicroBeta LumiJET 2460 Microplate Counter (Perkin Elmer, MA, USA) and compared to respective controls.

### 2.6. RNA Isolation and cDNA Synthesis

Total RNA isolation was performed using QIAzol lysis reagent (Qiagen, Hilden, Germany). RNA concentration was determined using NanoDrop-1000 (Thermo Fisher Scientific, MA, USA) and a minimum of 300 ng RNA was reverse-transcribed into cDNA with SuperScript III reverse transcriptase (Invitrogen, CA, USA) using a GeneAmp PCR System 9700 (Thermo Fisher Scientific, MA, USA).

### 2.7. Gene Expression Analysis

Amplification reactions were performed using a TaqMan Universal PCR mix (Thermo Fisher Scientific, MA, USA) on a 7500 Fast Real-Time PCR system (Applied Biosystems, CA, USA) with TaqMan probes (Supplementary Table S2). Gene expression was analyzed using the delta-delta Ct method (2−ΔΔCt) with genes of interest normalized to appropriate housekeeping genes.

### 2.8. Donor Genotyping

PHH and NPCs donors were genotyped for three genetic polymorphisms commonly associated with aberrant liver fat accumulation and risk of chronic liver disease [6]. Genomic DNA was isolated using a DNeasy Blood & Tissue Kit (Qiagen, Hilden, Germany). Genotyping for TM6SF2-E167K (rs58542926), PNPLA3-E434K (rs2294918), and PNPLA3-I148M (rs738409) was performed by TaqMan assay (Supplementary Table S2) using the 7500 Fast Real-Time PCR System (Applied Biosystems, CA, USA).

### 2.9. Immunohistochemistry

Spheroids were fixed overnight at 4 °C in 4% paraformaldehyde. Cryoprotected spheroids were embedded in Tissue-Tek OCT compound and sectioned at 8 μm. Slides were stained for: Albumin, CD68, COL1A1, CYP3A4, CYP2E1, α-smooth muscle actin (α-SMA), von Willebrand Factor (vWF), platelet-derived growth factor receptor β (PDGFRβ), TGFβ1, and vimentin (Supplementary Table S3). Secondary antibody staining was performed using a goat anti-rabbit Alex Fluor 488 and donkey anti-mouse Alexa Fluor 555. Slides were mounted with ProLong Gold Antifade Mountant with DAPI (Life Technologies, MA, USA), imaged using a Zeiss LSM710 confocal microscope (Zeiss, Oberkochen, Germany) or an Olympus IX73 inverted microscope (Olympus, Tokyo, Japan) and processed using Zen 2.5 blue edition analysis and ImageJ software. 

### 2.10. Statistical Analysis

Quantitative data was analyzed using GraphPad Prism version 5 (GraphPad Software, CA, USA) and described as the mean and standard error of the mean (SEM). Where feasible, donor combinations were assessed in replicates, for additional information on replicates see Table 2. Cell viability was determined for a minimum of 6 individual spheroids, RT-PCR was conducted using a pool of 24 spheroids, and immunohistochemistry was representative of a minimum of 6 imaged spheroids. Statistical analysis of differences induced by stimuli or drug screening was conducted using an unpaired, two-sided, non-parametric t-test comparing each drug to control only.

### 2.11. Ethical Approval

Primary human hepatocytes were obtained from commercially available sources and required no ethical approval by Karolinska Institutet. Ethical approval for the donors received from the project partners, at KaLy Cell, was obtained. Copies of documentation from Lonza and BioIVT regarding written consent by donors was obtained from the respective companies.

## 3. Results

In order to develop a human liver spheroid system suitable for modeling liver disease, we characterized the properties of PHH and NPCs from several different donors with varied demographic profiles. In addition, all donors were genotyped for mutations in the *TM6SF2* and *PNPLA3* genes, as shown in Table 1.

### 3.1. Human Liver Spheroids Stably Express Hepatocyte Markers

**Figure 1:**
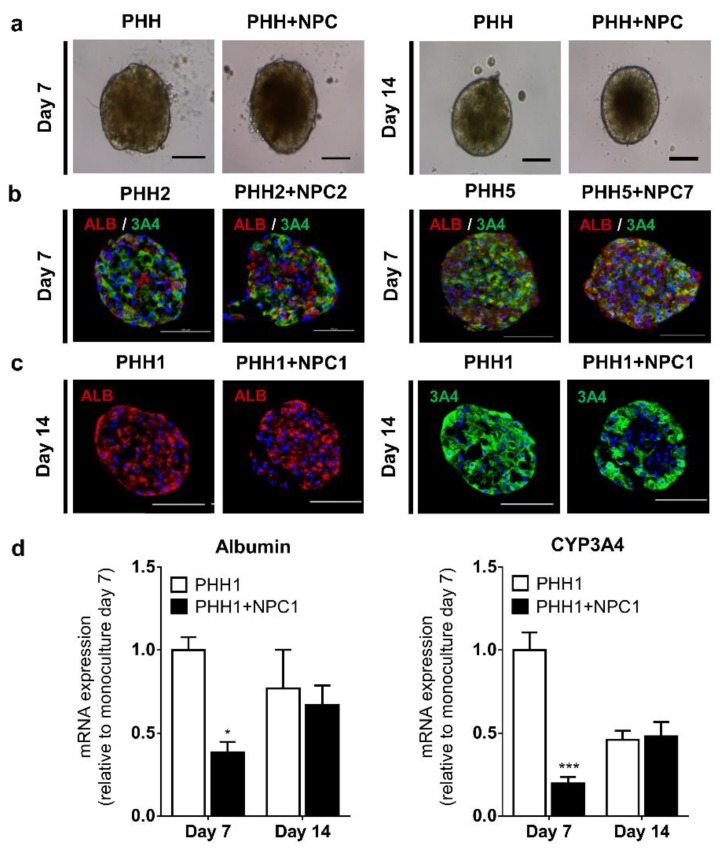
Hepatocyte markers in monoculture and co-culture spheroids. Typical spheroid morphology was maintained across monoculture and co-culture spheroids (**a**) over 14 days. Protein expression of albumin and CYP3A4 in monoculture and co-culture spheroids from different donors was evident at day 7 (**b**). Monoculture and co-culture spheroids expressed comparable albumin and CYP3A4 protein at day 14 (**c**). mRNA expression of albumin and CYP3A4, using the same donor (n = 3), at day 7 and 14 (**d**). ALB: albumin, 3A4: CYP3A4. * *p* < 0.05, *** *p* < 0.01.

Spheroid PHH monocultures and co-cultures maintained the same morphology (Figure 1a) and comparable ATP content (data not shown) over 14 days. The spheroid monocultures have previously been shown to exhibit a proteome and metabolome very similar to the freshly isolated hepatocytes from the same donors [18,19]. Here, we focused on the expression of the major hepatic proteins, CYP3A4 and albumin, which were found to be abundantly expressed in monoculture and co-culture spheroids for at least 14 days (Figure 1b–d). However, an initial decrease in albumin and CYP3A4 content was seen in the co-cultures, most probably caused by a delay in the re-differentiation of the hepatocytes during spheroid formation [19] in the presence of NPCs.

### 3.2. Liver Non-Parenchymal Cells Are Integrated in Human Liver Spheroids

**Figure 2:**
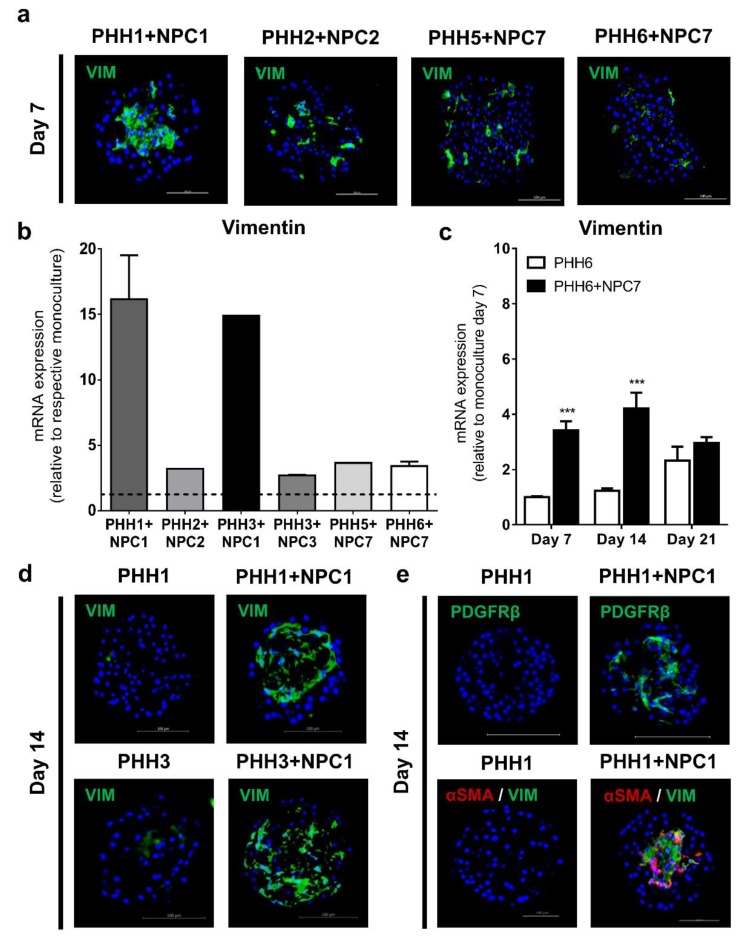
Spheroid co-cultures express markers of NPC populations. Co-cultures stained with vimentin show dispersed localization of HSCs in different spheroid co-cultures at day 7 (**a**). The mRNA expression of vimentin was very high in co-cultures containing NPCs from donor 1 (**b**). Incorporation of vimentin-expressing cells originated from the NPC fraction with the increase in vimentin mRNA expression from the same donor combination (n = 6) being highly reproducible over the time course (**c**). Incorporation of NPCs from donor 1 was independent of the PHH donor (**d**). The presence of HSCs was further validated with the use of PDGFRβ staining (**e**) and co-staining of vimentin and αSMA on day 14, suggesting an activated HSC phenotype exclusively observed in co-cultures (**e**). VIM: Vimentin, αSMA: alpha-smooth muscle actin. PDGFRβ: platelet-derived growth factor receptor β. *** *p* < 0.001.

Monoculture and co-culture spheroids, which were morphologically indistinguishable, were assessed for the presence of the different cell types in the NPC fraction. We observed that PHH monoculture spheroids expressed low levels of vimentin, a mesenchymal-derived HSCs [37] marker (Supplementary Figure S2a). However, vimentin-expressing cells were incorporated to varying degrees by all donors, evidenced by increased mRNA and protein expression in all co-culture spheroids (Figure 2a,b). The origin of these vimentin-expressing cells was primarily the NPC fraction, as determined by mRNA expression analysis of PHH and NPCs alone (data not shown), with biological replicates (n = 6) of PHH6 and NPC7 showing reproducible vimentin expression over time (Figure 2c). The incorporation of vimentin-expressing cells was similar regardless of whether or not matching PHH and NPCs from the same donor were used (Figure 2d). Furthermore, the identity of these cells as HSCs was confirmed by protein expression of PDGFRβ, which was only evidenced in co-cultures (Figure 2e and Supplementary Figure S2b,c). In human liver co-culture spheroids, endogenous HSCs activation was observed, as revealed by staining for αSMA, which localized with vimentin (Figure 2e). NPCs from donor 1 incorporated approximately 4-times more HSCs than any other NPC donors, having a potentially profound impact on the phenotype and consequent pro-fibrotic phenotype. 

Spheroid co-cultures were then assessed for vWF and platelet endothelial cell adhesion molecule (PECAM1) as well as CD68 as a markers of endothelial and Kupffer cells, respectively. Spheroid co-cultures expressed vWF and PECAM1 in both matched and non-matched configurations (Supplementary Figure S2d,e). However, a time-dependent increase in CD68 expression, which co-localized with vimentin-positive cells, was observed in both monoculture and co-culture spheroids (Supplementary Figure S3a–d). Both fibroblasts [38] and activated HSCs from human cirrhotic livers [39] can express CD68. Therefore, we assessed both CD14 and Toll-like receptor 4 (TLR4) mRNA expression as markers of monocyte-macrophage lineages and observed no difference between monocultures and co-cultures (Supplementary Figure S3e), suggesting the absence of macrophage lineages in co-cultures once spheroids have formed at day 7. The integration of the NPCs, some of which were not specifically investigated (lymphocytes, biliary cells), resulted in co-culture spheroids having improved mRNA IL-6 expression in response to non-toxic LPS stimulation (Supplementary Figure S4a) compared to monoculture counterparts (data not shown). These data indicate that human liver spheroids integrate different functionally active NPC populations and maintain expression of HSCs which are critical for development of NASH/fibrosis pathophysiology.

### 3.3. TGFβ Signaling Influences the Fibrogenic Response of Human Liver Spheroids

**Figure 3:**
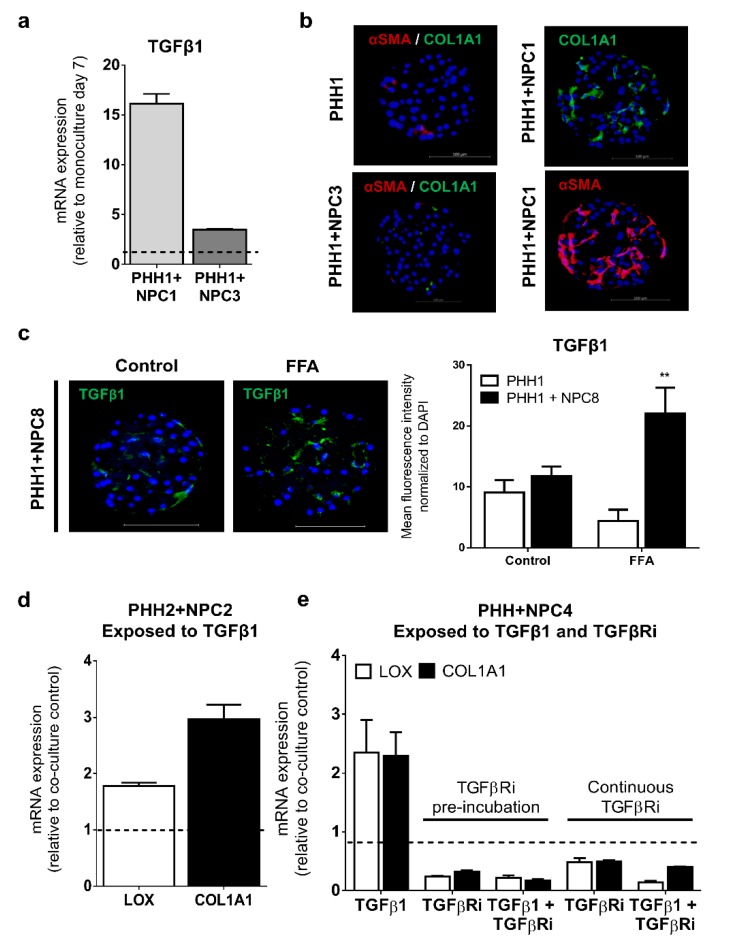
Reliance on TGFβ1 expression in NPCs to produce fibrogenic outcomes. NPCs from donor 1, as compared to donor 3 NPCs, when both combined with PHH1, had higher endogenous mRNA expression of TGFβ1 (**a**) which resulted in spontaneous protein expression of αSMA and COL1A1 (**b**). Additionally, TGFβ1 protein expression was typically higher in co-cultures (donor-dependent) and could be increased under FFA pressure (**c**). Exogenous TGFβ1 in co-culture spheroids from donor 2 induced COL1A1 and LOX mRNA expression (**d**) while in donor 4, similar induction was observed under TGFβ1 pressure, which was negated by TGFβRi added before or during cultivation (**e**). ** *p* < 0.01.

Given the importance of TGFβ signaling in chronic liver diseases [40], we examined the effect of TGFβ1 in the human liver spheroids. We observed that spheroids formed using certain NPC donors, which were more abundant in vimentin expression (NPC1), had high endogenous mRNA levels of TGFβ1 (Figure 3a), apparently providing the propensity for spontaneous fibrous tissue deposition, marked by progressively increasing COL1A1 and αSMA expression (Figure 3b). This demonstrated the reliance of TGFβ1 expression derived from the NPC population for adverse fibrogenic outcomes. Baseline TGFβ1 protein expression was assessed in monoculture and co-culture spheroids with exogenous stress caused by the addition of FFA increasing TGFβ1 proteins in co-cultures only (Figure 3c), which demonstrated mimicry of in vivo pathogenesis. 

Acute exposure (48–72 h) to non-toxic (Supplementary Figure S4b) exogenous TGFβ1 (5 ng/mL) induced LOX and COL1A1 mRNA expression, exemplified in donor 2 co-cultures (Figure 3d). These elevations of LOX and COL1A1 were inhibited by a TGFβ1 receptor (ALK5) inhibitor (TGFβRi), under either a 1 h pre-exposure to the TGFβRi prior to TGFβ1 stimulus or concurrently for the duration of acute exposure (Figure 3e).

**Figure 4:**
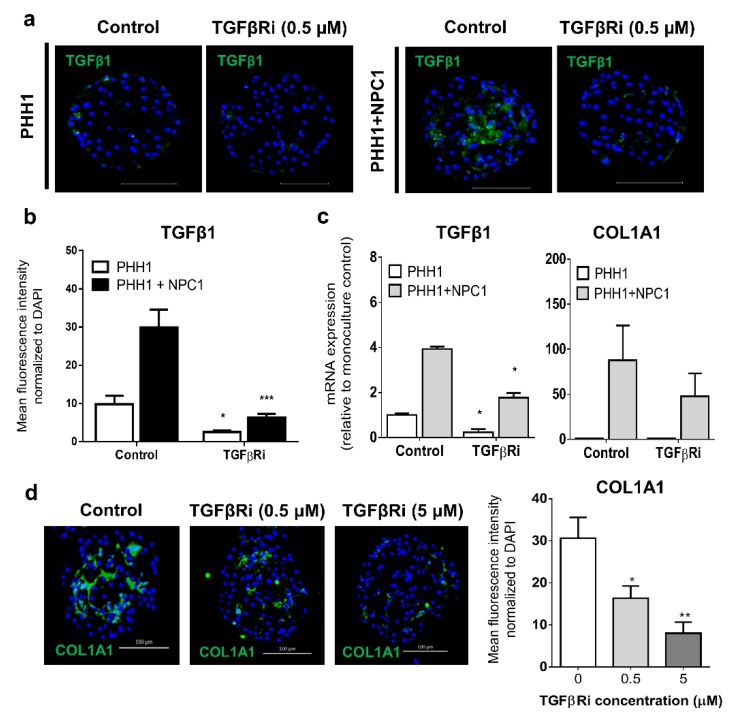
Regulation of fibrogenic outcomes by a TGFβ1 receptor inhibitor. Endogenous protein expression of TGFβ1 was high in donor 1 co-cultures but reduced following 72 h exposure to a TGFβRi (**a**, **b**) with a similar impact evident on mRNA expression of TGFβ and COL1A1 in co-cultures from donor 1 (**c**). The continuous exposure of TGFβRi for 7 days inhibited COL1A1 protein expression in a dose-dependent manner (**d**). * *p* < 0.05, ** *p* < 0.01.

Not all co-cultures responded to exogenous TGFβ1, which might be inherent in the fact that fully activated myofibroblasts which differentiate from the proto-myofibroblast lineage, have reduced sensitivity to TGFβ1 [7], resulting in co-culture responses varying with the degree of spontaneous versus induced activation stress. TGFβ1 expression in donor 1 spheroids differed markedly between monocultures and co-cultures, but was reduced at both protein (Figure 4a,b) and mRNA level (Figure 4c) by the addition of TGFβRi for 72 h. This modulation was reproducibly translated into a concentration-dependent decrease in COL1A1 protein expression following 7 days of exposure (Figure 4d), again demonstrating that inhibition of TGFβ1 action in spheroids reduced fibrogenic outcomes. NPCs from donors expressing high endogenous mRNA levels of TGFβ1 and COL1A1 produced a pro-fibrotic phenotype in the absence of exogenous stress. In addition, the expression of COL1A1 and αSMA protein increased differently over time in PHH–NPC combinations (data not shown), indicating endogenous inter-donor variations in the NPC cell fraction. However, TGFβRi repressed TGFβ1 mRNA expression in donor cells during the spheroid formation phase (Supplementary Figure S5a) despite consistent vimentin expression between exposed and non-exposed co-cultures spheroids (Supplementary Figure S5b). Combined, these data demonstrate that the important role for TGFβ1 signaling in fibrosis pathogenesis can be mimicked and attenuated in human liver spheroids.

### 3.4. Human Liver Spheroids can Mimic Attributes seen in NASH Patients when Exposed to FFA

**Figure 5:**
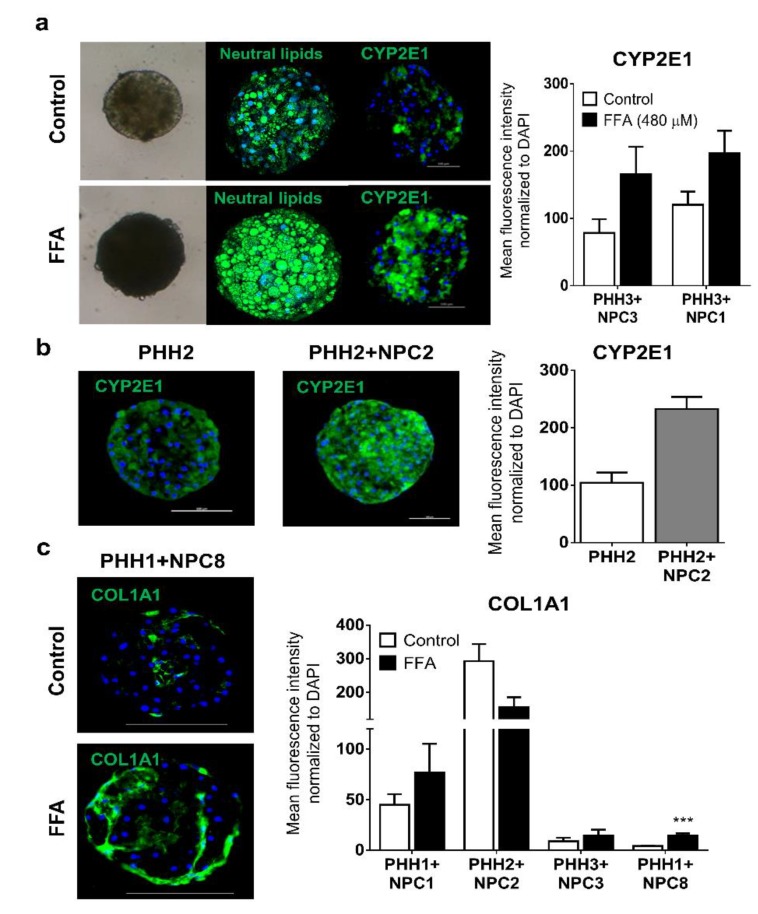
Free fatty acids induce lipid accumulation and can increase expression of CYP2E1, COL1A1, and αSMA. Lipid accumulation in spheroids exposed to FFA was evidenced by staining of neutral lipids, and an increase in CYP2E1 protein expression exemplified in donor 3 (**a**). CYP2E1 also increased in the presence of some NPCs (**b**). Increased COL1A1 protein expression under FFA pressure is exemplified at protein level in PHH1 + NPC8 and quantified across different donors, which demonstrated the influence of inter-individual variability in the baseline expression of these fibrogenic markers and ability to respond to FFA pressure (**c**). *** *p* < 0.001.

Abnormal accumulation of triglycerides in the liver is central in initiating dysregulation associated with NAFLD [41]. Steatosis can be induced in PHH monocultures [24,25], which was similarly achieved in co-culture spheroids exposed to non-toxic concentrations (Supplementary Figure S4c) of FFA for 8 days (Figure 5a). CYP2E1 metabolizes fatty acids and plays a key role in oxidative stress, and increased levels have been observed in the liver of NASH patients [42,43]. Indeed, exposure to FFA increased CYP2E1 mRNA (data not shown) and protein (Figure 5a) expression in co-culture spheroids. In some donors, co-culture spheroids exhibited elevated CYP2E1 protein expression in the presence of NPCs only (Figure 5b). Since CYP2E1 is a strong producer of reactive oxygen [44], the data suggests a contribution of ROS to the induced pathologies seen in the spheroids. 

The extent of fibrinogenesis induced in the spheroids differed between donors. Using the donor NPC1 and NPC2, each having mutations in the *PNPLA3* gene, high levels of spontaneous fibrogenesis was observed. However, under exogenous FFA exposure (480 µM), a pathophysiologically relevant increase in COL1A1 expression was observed in spheroids from PHH1 and NPC8, and to a lesser extent from matched donor 1 and donor 3 spheroids (Figure 5c). In contrast, for matched donor 2 spheroids, where an extremely high spontaneous COL1A1 expression was observed, no increase was observed by adding FFA. These data demonstrate that FFA can contribute to increased spheroid stress in co-culture spheroids where the spontaneous activation of NPCs is lower.

### 3.5. Human Liver Spheroids can be used as a Screening Platform for Anti-NASH Drugs

**Figure 6:**
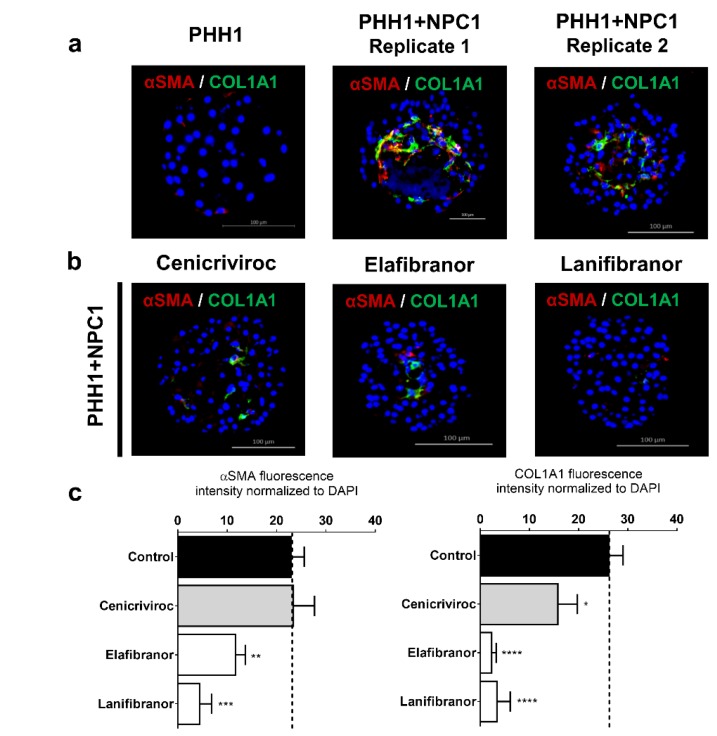
Effects of anti-NASH drugs on the attenuation of COL1A1 deposition in co-culture spheroids. Co-culture spheroids from donor 1 were reproducible in the fibrogenic phenotype (**a**). These spheroids were repeatedly exposed to different anti-NASH drugs, from day 7 to 14, with renewed drug exposure every alternate day. Expression of COL1A1 protein was reduced for cenicriviroc, elafibranor, and lanifibranor (**b**). Quantification (n = 10+) of αSMA and COL1A1 for each drug across replicate experiments (**c**). * *p* < 0.05 *, *** *p* < 0.001, **** *p* < 0.0001.

Co-cultures of PHH1 and NPC1, which spontaneously and reproducibly deposited fibrillary extracellular matrix exemplified by COL1A1 staining (Figure 6a), were used as a screening platform for anti-NASH/fibrosis drugs. The spheroids were repeatedly exposed to drugs from day 7 to day 14 of cultivation at a non-toxic concentration, as specified in Supplementary Table S1. Within this experimental context, extracellular fibrillary matrix deposition in co-culture spheroids was attenuated (Figure 6b,c) using the C-C chemokine receptor type 2 and type 5 antagonist cenicriviroc, the PPARα/γ/δ triple activator lanifibranor, and PPARα/δ agonist elafibranor. These results suggest that the spheroid system could constitute a valuable screening tool for active anti-NASH/fibrosis drug candidates based on the ability to reduce the deposition of COL1A1 and αSMA in this aggressively active phenotype.

## 4. Discussion

The multiple-hit hypothesis is the most accepted theory as to the onset of initiating NAFLD due to the heterogeneity of clinical outcomes [45], which implies that there is no single applicable way to model NAFLD in vitro. Here, we presented an HTS-compatible in vitro system where fibrogenic outcomes develop through the use of both endogenous and exogenous mechanisms in primary human liver spheroids. The inter-individual differences in phenotypic effects were demonstrated (Table 2) and provide a means whereby physiological, pathophysiological, mechanistic, and endocrine effects as the basis for such variability can be studied. Furthermore, since fibrotic phenotypes were efficiently observed and induced, this allowed for screening of anti-NASH/fibrosis compounds and may constitute a useful addition to future initiatives in this field when compared to other systems having been applied to investigate NAFLD and fibrosis, which do not make use of primary human tissue to generate hetero-cellular models [46,47].

Concepts integral to fibrosis progression include that oxidative stress drives hepatocyte damage and NPC activation resulting in fibrosis, TGFβ1 is a requirement for liver fibrosis, and that repression of TGFβ1 signaling can reduce fibrogenesis [11], which we were able to mimic in our liver co-culture spheroids. The foremost requirement for this system was to provide a biological context whereby known mechanisms of complex liver disease could be recapitulated in a reductionist in vitro setting.

In matched co-culture spheroids of donor 1, the mRNA expression of TGFβ1 increased during spheroid formation, which is consistent with the autocrine loop in TGFβ1 production associated with activated HSCs [48], which are the dominant contributor to collagen production [12]. This is also supported by the decreased TGFβ1 expression following the treatment of the spheroids with TGFβRi under comparable vimentin expression. TGFβ1 is considered the most potent pro-fibrogenic cytokine [48] and its contribution to the pathogenesis of fibrosis, targeting TGFβ1 ligands, has been considered for anti-fibrosis drugs [7].

PHH spheroids have been used to studying human genetic variants which contribute to altered lipid biosynthesis, and show promise for investigating clinically relevant associations in NAFLD [26]. As such, to improve the understanding of the underlying genetic landscape associated with enhanced predisposition to and progression of NASH/fibrosis, we genotyped for three clinically associated genetic polymorphisms [6] which could underpin some of the specific donor outcomes. CYP2E1 expression is increased in NASH patients [43] and later, clinical correlation was also established in children [49]. The role of CYP2E1 in NASH pathogenesis is supported in vitro via the impact of CYP2E1-derived ROS on stellate cell activation [50], by results of overexpression of CYP2E1 in mice [51] and in mice fed a high-fat diet [52]. In the spheroids, it was found that the FFA treatment, as well as the presence of NPCs, elevated the CYP2E1 expression both at the mRNA and protein level, which was most prominent in donor 2. The FFA effect was anticipated due to the capability of CYP2E1 to oxidize fatty acids [53].

Increased fibrosis in response to FFA was recapitulated. However, in most of the donors, the ability of FFA to be the catalyst for activation of HSCs was not observed, probably due to endogenously activated HSCs or due to donor-dependent lack of responses. Interestingly, increased incorporation of vimentin-expressing HSCs and a high baseline of extracellular fibrillary matrix was found in co-culture spheroids from donor 1 having the PNPLA3-I148M mutation. Under these matched donor conditions, we were able to detect attenuation of COL1A1 and αSMA deposition under clinically relevant conditions for the 3 drugs tested. The most effective drugs were the agonists of PPAR receptors lanifibranor and elafibranor, which have shown effects in animal models [54] and positive clinical effects in Phase IIb [8], respectively. Cenicriviroc, a chemokine 2 and 5 receptor antagonist, has shown both anti-inflammatory and anti-fibrotic effects in NASH patients and is now being tested in a Phase III clinical trial [35]. The inhibition of fibrosis in spheroids by these compounds, further indicates that the current spheroid system shows promising correlations to clinical data and should be further validated as a drug screening platform using donors with characterized genetic predisposition and titrated NPC composition.

We observed critical inter-individual variation across spheroid co-cultures in their capacity to respond to exogenous stimuli and exhibit a fibrogenic phenotype. The induction of fibrogenic outcomes were observed exclusively in co-culture spheroids with some NPC batches spontaneously producing fibrillary extracellular matrix accumulation over time, a phenomenon that was independent of a matching PHH and NPC phenotype and strongly reliant on the NPCs. We would propose that establishing a HTS-systems for NASH requires insight into the donor genotype, and furthermore, that due to the non-reliance on matched PHH–NPCs, the role underlying genetics of PHH or NPC donors can be controlled for and independently investigated for their impact on NASH.

## 5. Conclusions

Here, we established that this in vitro spheroid system sufficiently displays characteristics of a NASH phenotype which were attenuated when exposed to drugs in clinical development indicated for fibrosis. Therefore, this system, provided appropriate donor selection, is considered to be a valuable platform for both acute and chronic target-independent screening of compounds having anti-NASH properties.

## Figures and Tables

**Table 1:** Primary human hepatocyte and non-parenchymal cell donors.

Donor	PHH	NPC	Origin	Cultured NPCs	Genotype	Age	Gender	Ethnicity
Donor 1	X	X	KLC	Yes	Heterozygous PNPLA3-I148M	47	Female	Caucasian
Donor 2	X	X	KLC	Yes	Homozygous PNPLA3-E434K	61	Male	Caucasian
Donor 3	X	X	KLC	Yes	Heterozygous PNPLA3-I148M	25	Female	Caucasian
Donor 4	X	X	KLC	Yes	No polymorphisms	86	Male	Caucasian
Donor 5	X		BioIVT	n/a	No polymorphisms	30	Female	Caucasian
Donor 6	X		BioIVT	n/a	Heterozygous TM6SF2-E167K	22	Male	Caucasian
Donor 7		X	BioIVT	Unknown	Homozygous PNPLA3-I148M	Unknown	Female	Unknown
Donor 8		X	Lonza	Yes	No polymorphisms	55	Female	Caucasian

**Table 2:** Experiments conducted with various donor combinations and the outcomes.

Donor	Baseline Fibrillary Matrix	2xFFA and/or 3xFFA	Exogenous TGFβ	LPS	ALK5 Inhibitor (TGFβRi)
PHH1+NPC1	High	Mild increase in COL1A1 mRNA and protein expression ^#^	Minor increases in COL1A1 mRNA and protein expression ^#^	Increased IL-6 ^#^	TGFβ, LOX and COL1A1 mRNA expression reduced ^#^ Significantly reduced COL1A1 protein deposition ^#^
PHH1+NPC3	Low	No increase in COL1A1 or αSMA protein expression ^#^	No increase in COL1A1 mRNA or protein expression *	ND	TGFβ, LOX, and COL1A1 mRNA expression reduced *
PHH1+NPC8	Interme-diate	Increase in COL1A and αSMA protein expression *	ND	ND	ND
PHH2+NPC2	High	Increased COL1A1 mRNA expressionsion ^#^	Increased COL1A1 mRNA expression No protein increase *	Increased IL-6 *	ND
PHH3+NPC1	High	No increase in COL1A1 or αSMA protein expression ^#^	No increase in COL1A1 mRNA or protein expression *	ND	TGFβ, LOX, and COL1A1 mRNA expression reduced *
PHH3+NPC3	Low	Minor increase in COL1A1 or αSMA protein expression ^#^	No increase in COL1A1 mRNA or protein expression ^#^	Increased IL-6 ^#^	TGFβ, LOX, and COL1A1 mRNA expression reduced *
PHH4+NPC4	Low	No increase in COL1A1 mRNA or protein expression ^#^	No increase in COL1A1 mRNA or protein expression *	No response *	TGFβ induced increase in LOX and COL1A1 mRNA expression attenuated by TGFβRi ^#^
PHH5+NPC7	Low	Increased COL1A1 mRNA expressionNo protein increase *	Increased COL1A1 mRNA expressionNo protein increase *	Increased IL-6 ^#^	ND
PHH6+NPC7	Low	Increased COL1A1 mRNA expressionNo protein increase ^#^	Increased COL1A1 mRNA expressionNo protein increase *	Increased IL-6 *	ND

* experiment with 1 biological replicate; # experiments with 2 or more biological replicates; ND: not determine.

## Supplementary Materials

The following are available online at https://www.mdpi.com/2073-4409/9/4/964/s1. Supplementary Figure S1: Titration of the NPC incorporation. Supplementary Figure S2: Expression of NPC markers. Supplementary Figure S3: Expression of macrophage-lineage markers. Supplementary Figure S4: Cell viability in response to various stimuli. Supplementary Figure S5: TGFβ signalling during the formation of co-culture. Table S1: Anti-NASH drugs, Table S2: TaqMan assay probes, Table S3: Immunohistochemistry antibodies.
