# Supplementary material for: Human Liver Spheroids as a Model to Study Aetiology and Treatment of Hepatic Fibrosis
Source: Cells. 2020 Apr 14;9(4):964. doi: 10.3390/cells9040964 (PMC7227007; doi:10.3390/cells9040964)
Supplement: Supplementary file 1 [file cells-09-00964-s001.pdf]

Supplementary Tables:

Table 1: Anti-NASH drugs

**Drug**	**Target**	**Clinical phase**	**Concentration**
Cenicriviroc	CCR2/CCR5 inhibition	III	3 μM
Elafibranor	PPARα/δ agonist	III	20 μM
Lanifibranor	PPARα/γ/δ agonist	II	20 μM

Table 2: TaqMan assay probes

**Gene marker**	**TaqMan probes**
Albumin	Hs00910225_m1
CD14	Hs02621496_s1
CD68	Hs02836816_g1
COL1A1	Hs00164004_m1
CYP2E1	Hs00559367_m1
CYP3A4	Hs00604506_m1
Interleukin-6	Hs00174131_m1
Lysyl oxidase	Hs00942483_m1
PDGFRβ	Hs01019589_m1
PECAM1	Hs01065282_m1
PNPLA3 E434K (rs2294918)	C_2520500_10
PNPLA3 I148M (rs738409)	C_72341_10
Tata-binding protein	Hs00427620_m1
Toll-like receptor 4	Hs00152939_m1
TM6SF2 E167K (rs58542926)	C_89463510_10
Transforming growth factor β1	Hs00998133_m1
Vimentin	Hs00958111_m1
α-smooth muscle actin	Hs00426835_g1

Table 3: Immunohistochemistry antibodies

**Primary antibody**	**Species**	**Vendor**	**Dilution**
Albumin	Mouse	Santa Cruz: Sc51515	1/200
CD68	Mouse	Santa Cruz: Sc-70761	1/50
COL1A1	Rabbit	Abcam: ab34710	1/200
CYP2E1	Rabbit	In-house	1/300
CYP3A4	Rabbit	Cypex: PAP011	1/5000
PDGFRβ	Rabbit	Abcam: ab32570	1/300
TGFβ1	Rabbit	Abcam: ab92486	1/200
Vimentin	Rabbit	Abcam: ab28364	1/100
von Willebrand factor	Rabbit	DAKO: A0082	1/200
α-smooth muscle actin	Mouse	Abcam: ab7817	1/200
**Secondary antibody**	**Species**	**Vendor**	**Dilution**
Alexa Fluor 488	Goat anti-Mouse IgG	Thermo Fisher: A-10680	1/500
Alexa Fluor 594	Goat anti-Rabbit IgG	Thermo Fisher: A-11037	1/500

Supplementary Figure legends:

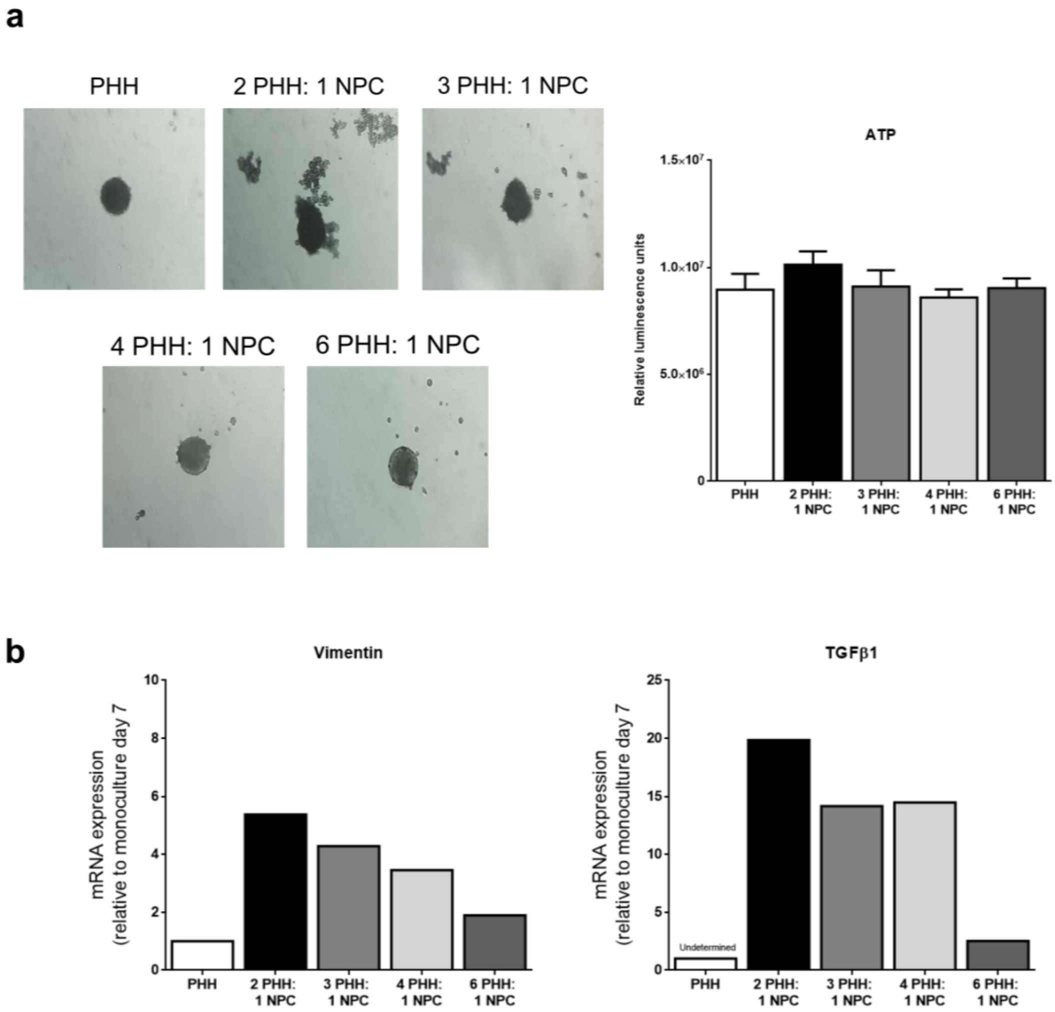

Supplementary Figure 1: Titration of the NPC incorporation. Non-parenchymal cells were added to a constant number of PHH at a ratio of 2:1, 3:1, 4:1, and 6:1 and morphology and viability (ATP content) observed for cellular integration (a). mRNA expression of the hepatic stellate cell marker, vimentin, was used to assess the fold-increase in NPC markers per ratio and the consequences for TGFβ1 expression also assessed (b).

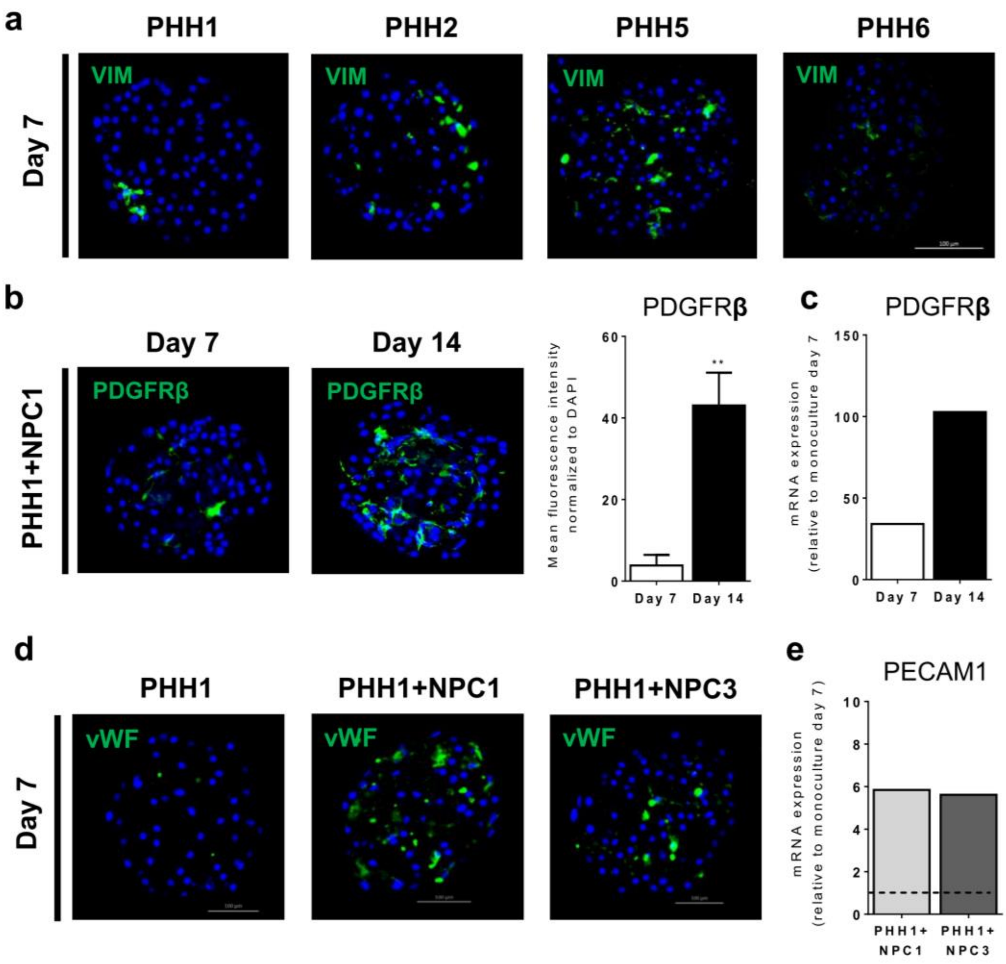

Supplementary Figure 2: Expression of NPC markers. Expression of vimentin in spheroid monocultures of different donors (a). PDGFRβ protein confirmed the presence of HSCs in the co-culture and increased over the time-course from day 7 to day 14 (b). The PDGFRβ expression was also confirmed at the mRNA level and was much higher in co-as compared to mono-cultures (c). In addition, the co-cultures expressed LSECs as marked by vWF immunostaining (d) and PECAM1 mRNA expression (e) at day 7. ** p -value < 0.01.

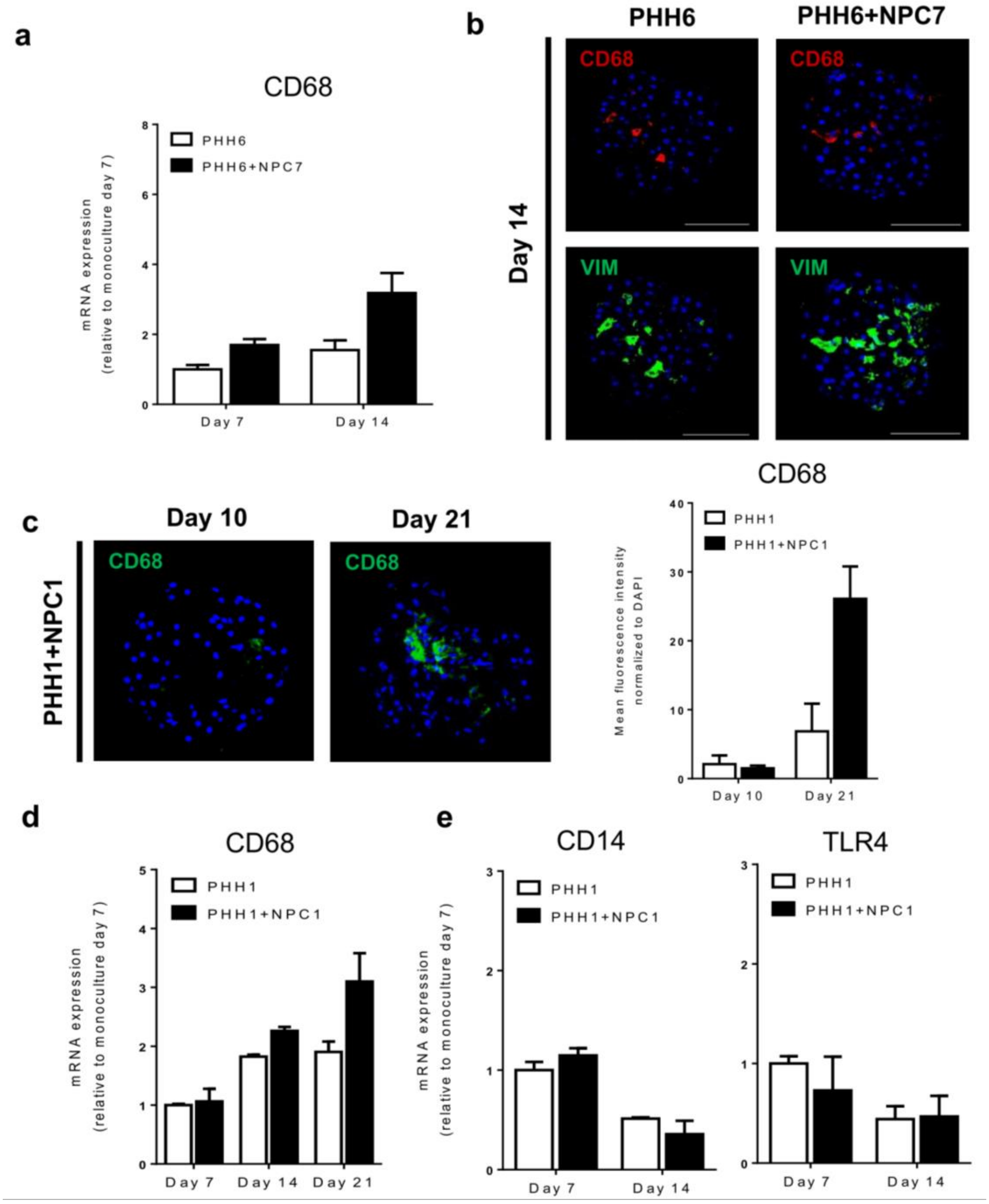

Supplementary Figure 3: Expression of macrophage-lineage markers. CD68, an antigen trafficking protein used as a marker of Kupffer cells, was evident at mRNA (a) and protein level (b). However, in both monoculture and co-culture spheroids the staining co-localized with vimentin positive cells and increased over time, suggesting that the CD68 was being expressed in myofibroblast lineages. Similar increases in CD68 protein were observed over time in other donor co-cultures (c-d). To confirm the presence or absence of macrophage-lineages in co-culture spheroids, CD14 and TLR4 were assessed but displayed no difference between monoculture and co-culture spheroids (e) suggesting the absence of macrophage lineages in co-cultures at day 7 once spheroids have formed.

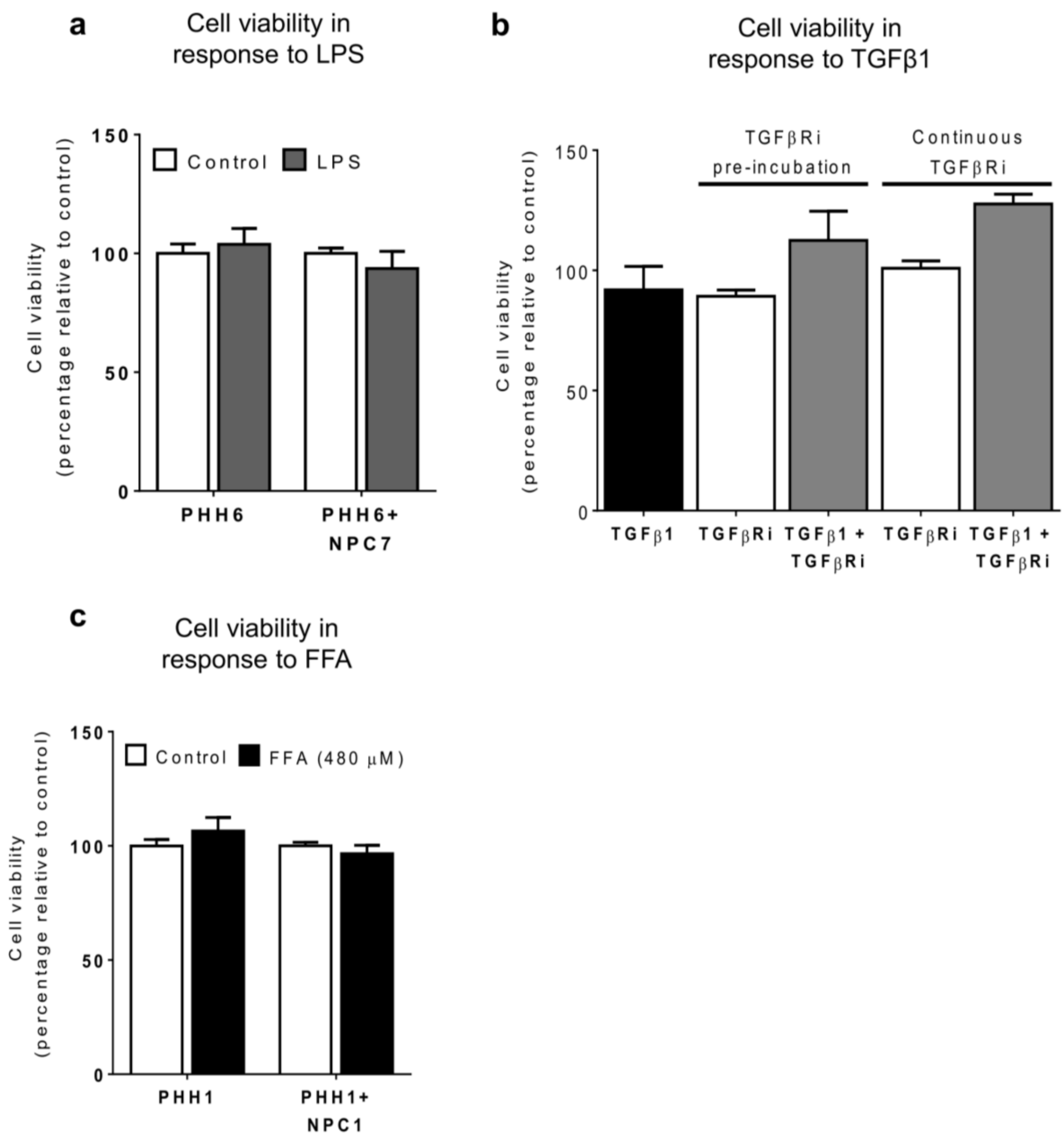

Supplementary Figure 4: Cell viability in response to various stimuli. Exposure to LPS (a), TGFβ1 in donor 4 (b), and FFA (c) did not have detrimental effects on spheroid viability.

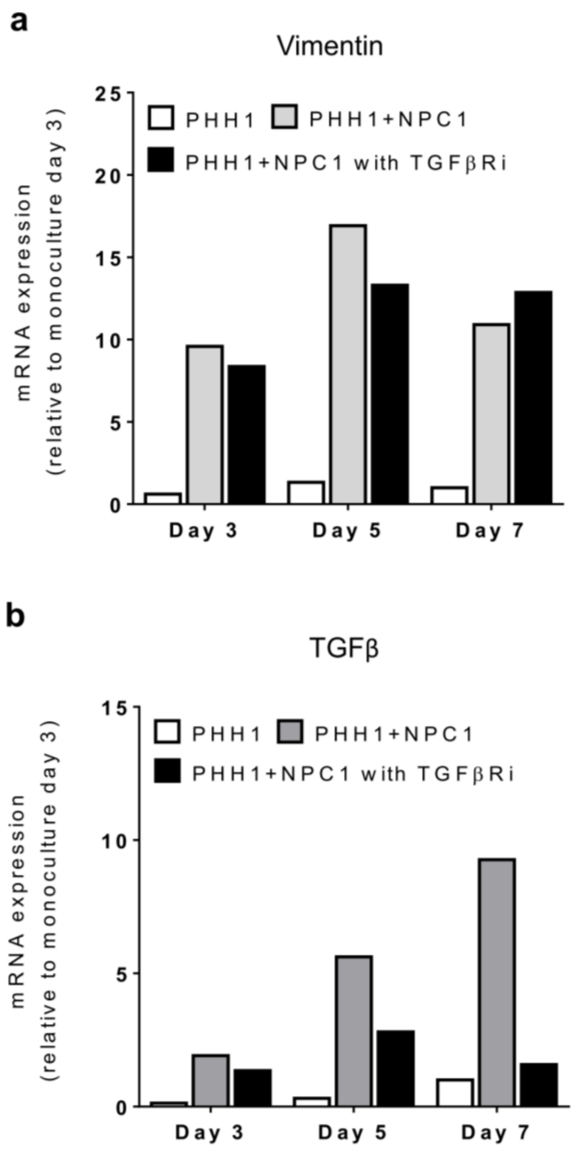

Supplementary Figure 5: TGFβ signalling during the formation of co-culture spheroids. Co-cultures of donor 1 expressed higher vimentin mRNA (a) corresponding with an increased expression of TGFβ mRNA which gradually increased during spheroid formation but could be attenuated by TGFβRi (b).
